# Pleiotropic Roles of CXCR4 in Wound Repair and Regeneration

**DOI:** 10.3389/fimmu.2021.668758

**Published:** 2021-05-28

**Authors:** Huating Chen, Gongchi Li, Yiqiong Liu, Shuaifei Ji, Yan Li, Jiangbing Xiang, Laixian Zhou, Huanhuan Gao, Wenwen Zhang, Xiaoyan Sun, Xiaobing Fu, Binghui Li

**Affiliations:** ^1^ Department of Wound Repair Surgery, Institute of Geriatric Medicine, Liyuan Hospital, Tongji Medical College, Huazhong University of Science and Technology, Wuhan, China; ^2^ Research Center for Tissue Repair and Regeneration Affiliated to the Medical Innovation Research Department and 4th Medical Center, PLA General Hospital and PLA Medical College; PLA Key Laboratory of Tissue Repair and Regenerative Medicine and Beijing Key Research Laboratory of Skin Injury, Repair and Regeneration; Research Unit of Trauma Care, Tissue Repair and Regeneration, Chinese Academy of Medical Sciences, Beijing, China; ^3^ Department of Southern Hospital of Southern Medical University, Southern Medical University, Guangzhou, China; ^4^ Department of School of Biological Engineering, Chongqing University, Chongqing, China; ^5^ Chongqing University, Chongqing, China

**Keywords:** CXCR4, wound healing, inflammation, proliferation, adhesion, chemotaxis, apoptosis

## Abstract

Wound healing is a multi-step process that includes multiple cellular events such as cell proliferation, cell adhesion, and chemotactic response as well as cell apoptosis. Accumulating studies have documented the significance of stromal cell-derived factor-1 (SDF-1)/C-X-C chemokine receptor 4 (CXCR4) signaling in wound repair and regeneration. However, the molecular mechanism of regeneration is not clear. This review describes various types of tissue regeneration that CXCR4 participates in and how the efficiency of regeneration is increased by CXCR4 overexpression. It emphasizes the pleiotropic effects of CXCR4 in regeneration. By delving into the specific molecular mechanisms of CXCR4, we hope to provide a theoretical basis for tissue engineering and future regenerative medicine.

## Introduction

**Figure 1:**
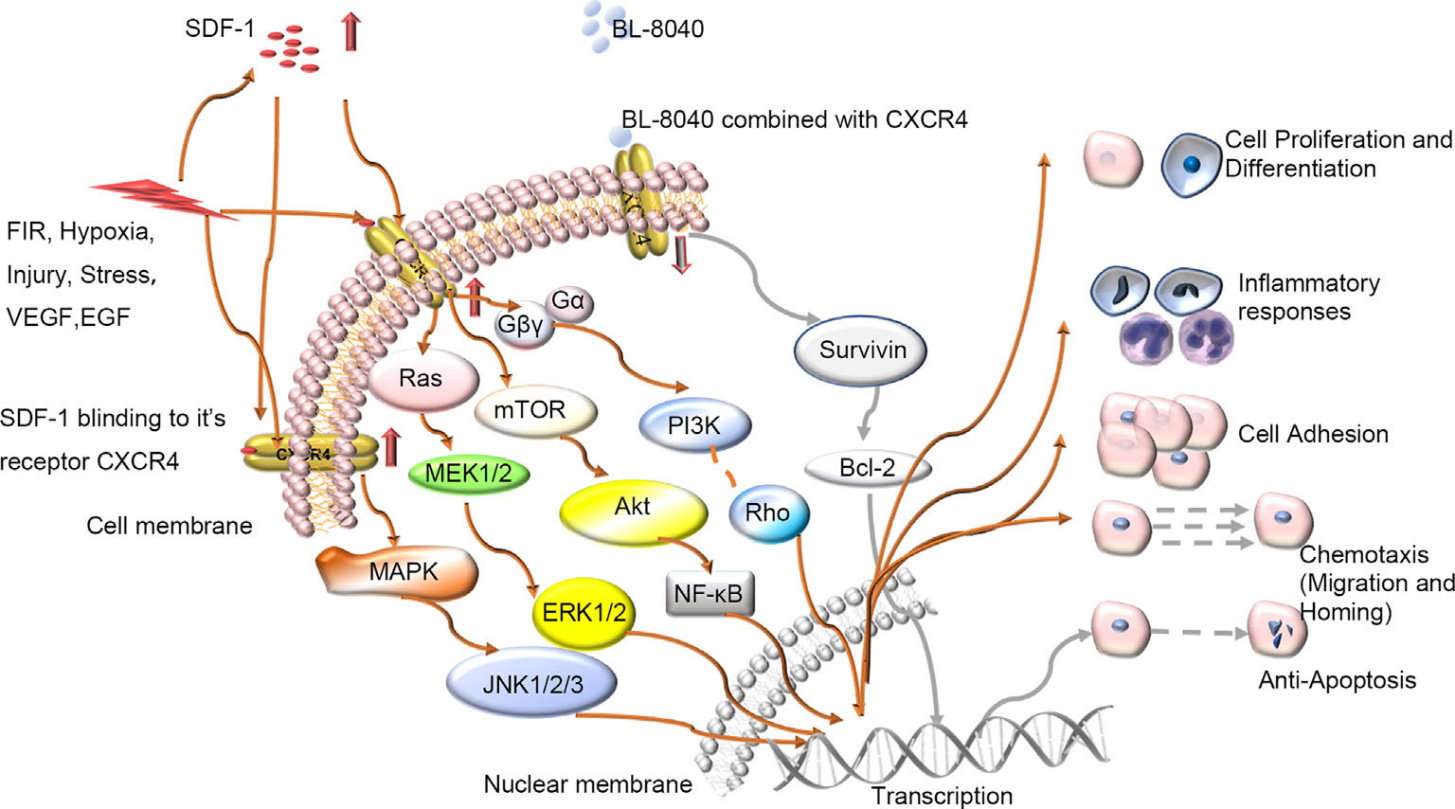
Schematic diagram of intracellular signal transduction pathways related to wound healing involving SDF-1/CXCR4. Various stimuli *in vivo* and *in vitro* will affect the expression of CXCR4 on the cell membrane, regulate the CXCR4 signaling pathway and participate in various processes of wound repair including proliferation, differentiation, and migration etc. BL-8040: antagonist of CXCR4; FIR, Far-infrared radiation; VEGF, vascular growth factor; EGF, epidermal growth factor.

The skin wound-healing process is complex and dynamic but highly carefully arranged, with intersecting sequence events between phases. It involves many kinds of cells and factors. C-X-C chemokine receptor 4 (CXCR4) is one of the most important. CXCR4 can be bound by stromal cell-derived factor-1(SDF-1), CD4 and CD74, and SDF-1 may be the only endogenous ligand of CXCR4 (1). CXCR4 plays a pivotal role in both physiological processes such as germ cell development (2), neurogenesis (3), vascular formation (4) and cardiogenesis (5) and pathological processes such as muscle regeneration (6, 7) and vascular formation (8). CXCR4 can be upregulated during injury, hypoxia, stress and vascular tissue damage (9). When tissues such as the brain (10), heart (11), kidney (12), and liver (13) are damaged, the secretion of SDF-1 can significantly increase. With wounding, CXCR4-positive stem cell/precursor cells are induced to express early tissue markers in bone marrow and participate in wound repair and regeneration. The SDF-1/CXCR4 axis can activate the major physiological processes associated with wound healing such as chemotaxis of inflammatory cells to damaged tissues (14–18), cell proliferation for wounds repair (19–23), and collagen deposition for tissue remodelling (24). The activated SDF-1/CXCR4 signaling pathway in turn activates several signaling pathways, including phosphoinositide 3 kinase (PI3K)/protein kinase B (PKB) (also known as AKT, PI3K/AKT), mammalian target of rapamycin (mTOR), and Janus kinase/signal transduction and transcription activator pathways as well as nuclear factor-activated light chain enhancer B cells (NF-κB) involved in regulating intracellular transcription, Ca2+ efflux, and cell survival (25) (**Figure 1**). Thus, the SDF-1/CXCR4 signal axis can transduce multiple signals to control the biological functions of cell survival, proliferation, chemotaxis, apoptosis and differentiation (26), and enhance angiogenesis in targeted diseases (8, 27, 28). So, it plays an important role in wound healing.

A previous quantitative trait loci mapping study (29) linked SDF-1 to regenerative capacity (14). Overexpression of CXCR4 improves cell engraftment and survival as well as limb salvage and tissue regeneration after injury (30). In addition, the SDF-1/CXCR4 axis can enhance the activation of endogenous tissue repair pathways (29). Different strategies have been tried to promote tissue repair by increasing SDF-1 availability to improve the regeneration of intervertebral discs(IVD) (24), muscles (7), and liver (31) (**Figure 3**). Several regeneration experiments that rely on overexpressing CXCR4 tissue have achieved great success in mice and humans (6, 8, 24, 32–34). Guo et al. (32) found that the level of SDF-1 around the wound edge increased significantly after injury, and blocking this signal axis *in vivo* delayed wound healing. Another team (33) found that the reduced form of high mobility group box 1 can orchestrate tissue regeneration in liver and muscle. Kim et al. (6). showed that CXCR4-overexpressing adipose tissue-derived stem cells (ADSCs) more efficiently contributed to muscle tissue regeneration than normal ADSCs in a diabetic mouse model. Wei et al. (24) showed that mesenchymal stem cells (MSCs) could overexpress CXCR4 (CXCR4-MSC), which enhanced their migration and improved the speed of IVD regeneration. Activated tissue-resident MSCs can also regulate the expression of CXCR4 on natural killer cells to promote the regeneration of vasculature (8). A study of traumatic brain injury reported that the transplantation system of human umbilical-cord MSCs and activated astrocytes could be used to repair moderate-sized lesions by activating CXCR4 (34). These observations suggest that the exploration of CXCR4 and its functions have brought insights into tissue regeneration engineering and that using appropriate methods to enhance CXCR4 signaling can improve tissue regeneration. However, the specific molecular mechanism is not yet fully understood, so further experimental exploration is needed.

## CXCR4 Contributes to Cutaneous Wound Healing

### SDF-1/CXCR4 Signaling Induces the Inflammatory Response During Wound Healing

**Figure 2:**
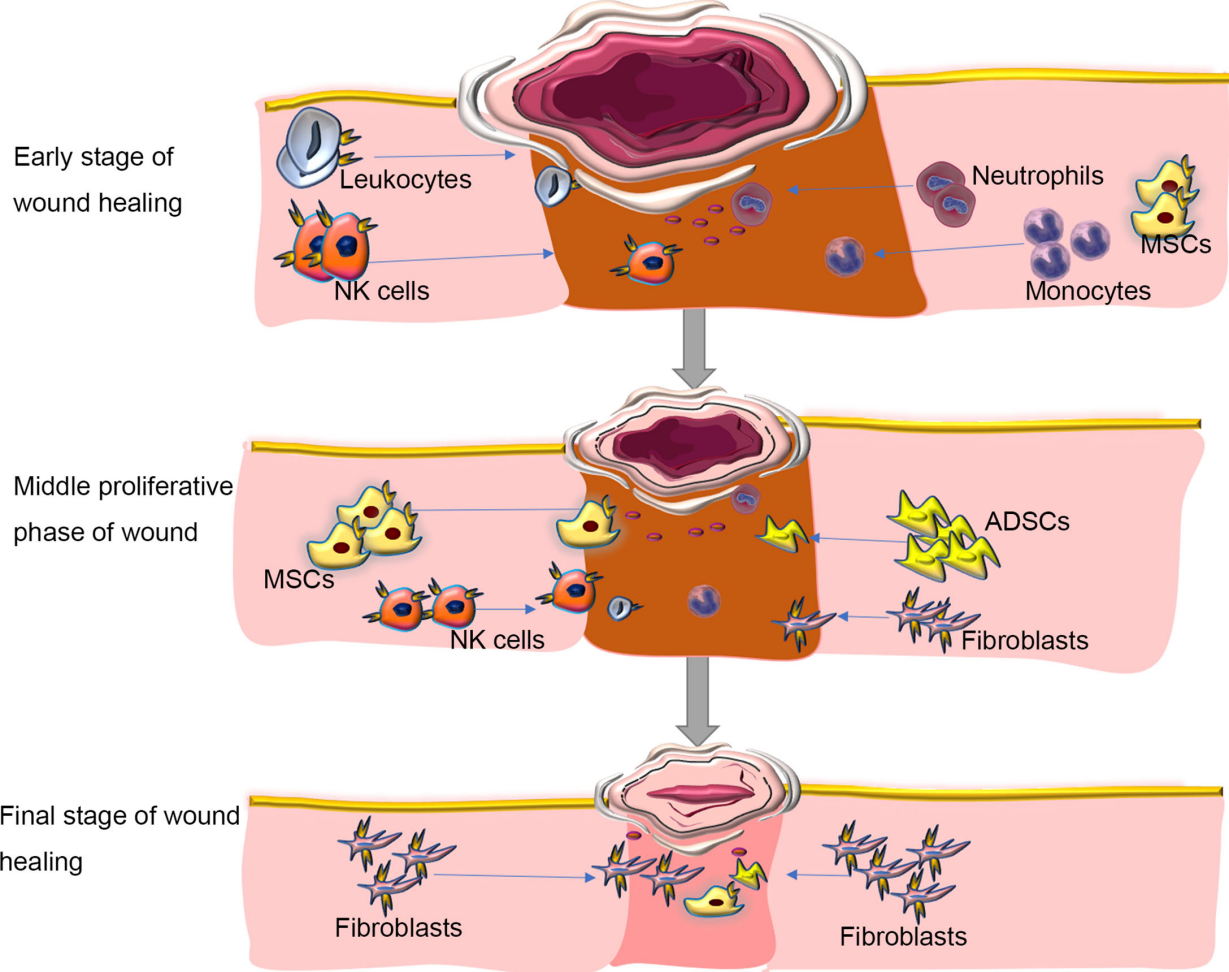
Roles of SDF/CXCR4 axis in regulating inflammation during wound repair. Inflammation is one of the major molecular and cellular events at the early stage of wound healing. CXCR4-expressing neutrophils can be first recruited in response to injury signals to the wound bed, followed by monocytes and Leukocytes to reinforce the inflammatory reaction. During inflammation, immune cells aggregated at the wound site, which can not only fight against invading microorganisms, but also produce various growth factors, such as FGF to direct re-epithelialization, fibroblast reconstruction and ECM remodeling. In the middle proliferative phase of wound healing, autologous or allogeneic stem cells are mobilized and migrate into the wound site, where they proliferate, secret cytokines, and participate in various cellular events in response to activation of SDF/CXCR4 signaling, including angiogenesis, muscle regeneration, and collagen synthesis. At the final stage of wound healing, immune cells progressively disappear. The fibroblasts deposit new extracellular matrix, which is gradually remodeled to form scar tissues. Blocking SDF/CXCR4 axis may result in a reduction in collagen production, thereby alleviating tissue fibrosis at the wound site. ADSCs, adipose tissue-derived stem cells; MSCs, mesenchymal stem cells. NK Cells; Natural Killer Cells. Blue arrows showing the migration/homing of cells induced by the SDF-1/CXCR4 axis.

Because tissue damage is often accompanied by acute inflammation and tissue regeneration and the three are inevitably entangled, some experts suggest that regeneration cannot be achieved without inflammation (35). The primary function of inflammation is to eliminate the invasion of pathogens and to remove tissue necrosis, to maintain the homeostasis of tissue. Many studies have shown that CXCR4 is expressed on inflammatory cells and prime their migration ability to ischemic tissues, thereby participating in revascularization and tissue repair (14–18). In parallel, SDF-1, the ligand of CXCR4, is constitutively expressed in specific lymphoid or nonlymphoid tissues (16) and thus participates in inflammation. It seems that neutrophils majorly contribute the CXCR4/SDF-1 necessary for wound healing. Neutrophils can be first recruited early during the inflammatory response (36). Some have suggested that during the acute inflammatory reaction, the first line of defense against invading pathogens is the CXCR4^hi^ neutrophil subsets, which rapidly migrate to the site of inflammation (17, 18, 37–39), followed by monocytes to reinforce the inflammatory reaction (37–39). Ageing neutrophils can upregulate the expression of CXCR4 on their surface, which allows them to return to the bone marrow, where they are eventually swallowed and eliminated by the resident macrophages (40), thus contributed to resolve wound inflammation. Another study found that in wild-type mouse models after tissue injury, SDF-1 is upregulated in the wound epidermis and recruits CXCR4-expressing leukocytes to the injury site (14). Then, leukocytes migrate into lesions to destroy invading microorganisms and clear debris (41)(**Figure 2**).

Essentially, wound treatment represents the regeneration of the epidermis and dermis but also the restoration of skin function (42). Typically, wound healing in adult mammals, especially humans, results in scar tissue without regeneration of skin appendages. A large area of skin scars will affect the appearance of the patient and can lead to functional dysfunction such as inability to sweat and dissipate heat, thereby affecting quality of life. We are aware, however, that inflammatory responses can be both protective and deleterious in wound healing. The initiation of the early wound healing response requires the recruitment of immune cells, but the suppression of some forms of immunity can accelerate the subsequent regeneration (43). In the process of inflammation, immune cells gather at the wound site, which can not only fight against invading microorganisms, but also produce various growth factors, such as FGF to guide re-epithelialization, fibroblast reconstruction and ECM remodeling (44) (**Figure 2**). At the same time, inflammation can limit regeneration by promoting fibrosis and scar formation, leading to related dysfunction. The SDF-1/CXCR4 axis can promote inflammation to cause fibrosis and scar formation (14, 45). The use of the CXCR4 inhibitor AMD3100 can significantly reduce the subsequent recruitment of CXCR4-expressing leukocytes and achieve scarless repair of skin wounds and appendages regeneration in mice (14). There are also reports that AMD3100 improved wound healing and scar formation in diabetic mice (46). Thus, regulating the expression of CXCR4 temporally and spatially can provide a new and feasible way for complete scarless healing in mammals and even humans during wound healing.

### CXCR4 Promotes Proliferation During Wound Healing

CXCR4 is involved in cell proliferation, which is the basis of tissue regeneration. After acute liver injury, SDF-1 and CXCR7 seem to play a major role in liver regeneration by promoting hepatocyte proliferation (19). A recent study *in vitro* showed that SDF-1 can improve the effect of cytokines on the clonal growth of normal myeloid progenitors (47). Also, SDF-1 may play a key role in epithelialization by promoting epidermal stem cell migration and proliferation (32). Pasha and others (48) proposed that the advancement of SDF-1a can enhance the survival, engraftment and proliferation of MSCs treated with SDF-1a/CXCR4 signaling in infarcted myocardium. Cell proliferation involves many signal axis pathways; among the numerous signal traffic networks, CXCR4 seems to play an important role. Many studies have shown that the CXCR4 signaling pathway is involved in cell proliferation (20–23). The mitogen-activated protein kinase (MAPK) pathway has also been implicated in mitogen-stimulated proliferation (20). CXCR4 binding to its ligand may lead to receptor internalization and MAPK activation to enhance cell proliferation (21). Another study suggested that SDF-1–induced cell proliferation works by activating extracellular signal-regulated kinase (ERK) (22). Moreover, SDF-1–induced ERK1/2 activation can be directly mediated by MAPK kinase 1/2 signaling with no need for synthesis of new proteins or Gαi participation (49). After acute liver injury, SDF-1 and CXCR7 seem to play a major role in liver regeneration by promoting hepatocyte proliferation (19). Far-infrared radiation can upregulate CXCR4, Nanog, Sox2, c-Kit, Nkx2.5, etc. at the mRNA and protein levels to promote cell proliferation and migration; blocking CXCR4/ERK activation can prevent far-infrared radiation-induced cell proliferation and migration (23).

### CXCR4-Directed Cell Adhesion During Tissue Regeneration

Effective directional migration cannot work without adhesion (41), and it plays an important role in wound repair. Adhesion plays a role in cell survival, cell migration, inflammation, and angiogenesis, and apoptosis. These processes are essential for wound repair. The CXCR4 signal axis can affect cell adhesion by regulating the expression of adhesion molecules (50–58). SDF-1 can upregulate the expression of adhesion molecules such as very late activation antigen 4 (VLA-4[α4β1]), VLA-5, and lymphocyte function-associated antigen 1 (50–52), thereby increasing cell adhesion. Adhesion molecules have multiple ways of regulating wound healing. For example, adhesion molecule–ligand interactions are the initial process during the proper homing of hematopoietic stem cells to the bone marrow (52, 59). Cells can use β-integrins, VLA-4 and VLA-5 to bind to stromal layers, which is important for bone-marrow engraftment (55). Also, integrins such as VLA-4 can cooperate with chemokine receptors such as CXCR4 to promote the adhesion to MSCs (60). Adhesion of MSCs in turn favors cell survival and growth, proliferation, and tissue retention (60, 61). Alternatively, integrin–growth factor pairs contribute to angiogenesis *via* various signaling pathways, and integrins can be involved in cell survival or prime the process of apoptosis (62).

Adhesion molecules are closely related to cell migration. Cells polarize firstly before migrating. Extracellular matrix can connect to the intracellular cytoskeleton and alter cytoskeletal dynamics, with the help of integrins (63–65), thus playing a role in changing their cellular localization during cell polarization. In migrating cells, a number of adhesion molecules are concentrated in the uropod to promote the binding of other cells, thus enhancing the recruitment of leukocyte and migration of transendothelial (66). Adhesion molecule VLA-4 can be expressed on monocytes, lymphocytes and most other hematopoietic cells and plays an important role in lymphocyte trafficking and homing (67), which control matters for early stages of tissue repair. In addition, integrins are the main family of migration-promoting receptors that can significantly promote cell migration (41).

Meanwhile, with stimulation of SDF-1, VLA-4–mediated adhesion to fibronectin is increased and results in an increased overall adhesion (55). SDF-1 can not only increase integrin surface expression but also control adhesion molecules by enhancing integrin activation (52, 56). The cell adhesion effect regulated by SDF-1/CXCR4 signaling is mainly regulated by PI3K, MAPK and ERK signaling pathways, with PI3K playing a critical role. PI3K appears to be required for SDF-1α–mediated phosphorylation of focal adhesion proteins, whereas MAPK ERK1/2 is not (68). The activation of PI3K can lead to the phosphorylation of several focal adhesion components such as Crk-associated substrate, proline-rich kinase-2, focal adhesion kinase, paxillin, Crk-L, Crk, and Nck (69). PI3Ks can also regulate cell adhesion by phosphorylation of AKT (21). Other signaling pathways such as MAPK have also been reported. This adhesive interaction between cells and extracellular matrix of stromal cells increases the expression of β1, α3, α6, and αv integrins and increases tyrosine kinase activity, which in turn prevents caspase activation, thus resulting in decreased chemotherapy-induced cell death (70).

## CXCR4-Mediated Chemotaxis Is Critical to Migration

Chemotaxis is a response produced by organisms to chemical substances in the external environment. Cell chemotaxis allows cells to migrate to where the relevant chemicals are located and generally perform the corresponding biological function. Cell chemotaxis provides a basis for cell migration, which is also a prominent component of tissue repair. Chemokines and chemokine receptors provide directional cues for cell migration. The hallmarks of ligand-stimulated chemotaxis are rearrangement of the cytoskeleton, polymerization and polarization of actin formation, and adhesion of pseudopods (71), to promote the migration of cells. During wound repair, cell migration mainly occurs in immune response cells like lymphocytes and stem cells, such as MSC cells, which implicated in injury repair.

Migration is a prominent component of chemotaxis. The main role of chemokines is lymphocyte trafficking (**Figure 2**). Cell migration is a highly ordered customized multi-step process that is also a prominent component of various tissue repair and regeneration (41). The healing of chronic wounds requires the migration of stem cells to the diseased area to replace damaged or lost cells. Effectively inducing their migration to the lesion area is a problem that requires more investigation. The function of CXCR4 offers a potential research direction to solve this problem. SDF-1 at low levels is a chemoattractant for lymphocytes but at high levels becomes a chemical repellent (72). In T lymphocytes, SDF-1 appears to depend on PI3-kinases and Src tyrosine kinases to stimulate the activation or phosphorylation of Tec kinases Itk and Rlk (73, 74). A loss-of-function Itk mutant showed impaired induced migration to SDF-1 (73). Tyrosine kinase ZAP-70 may work to enhance the migration of SDF-1; when not present in cells, the migration to SDF-1 is reduced (75). CXCR4 receptor was detected and expressed on progenitor cells and inflammatory cells, which can promote their migration to ischemic tissues, thereby participating in blood remodeling and tissue repair (15).

Stem cell-directed migration to targeted tissues is called homing. CXCR4 and its ligand SDF-1 are among the most important chemokines of stem cells (76). The activation of SDF-1/CXCR4 can induce the migration of stem cells to repair damaged tissue (77–79) and promote wound healing. CXCR4 and its ligand SDF-1 constitute the most studied chemokine–chemokine receptor axis in MSC homing (80). CXCR4 can play an important role in the migration of pre-existing or externally transplanted stem cells to the damaged site (81, 82). In addition, MSCs overexpressing CXCR4 can enhance migration to SDF-1, shown through chemotaxis experiments *in vitro* (24). The interaction between transplanted hematopoietic stem cells and bone-marrow endothelial cells and migration through the endothelium is the first step for hematopoietic stem cells to home properly to the bone marrow (52, 59). Kim et al. (6) considered that the homing and engraftment of ADSCs is related to the transfection efficiency of CXCR4 because transplantation of ADSCs overexpressing CXCR4 into the ischemic area of rats significantly increased ADSCsCXCR4+ homing and engraftment (**Figure 2**).

**Figure 3:**
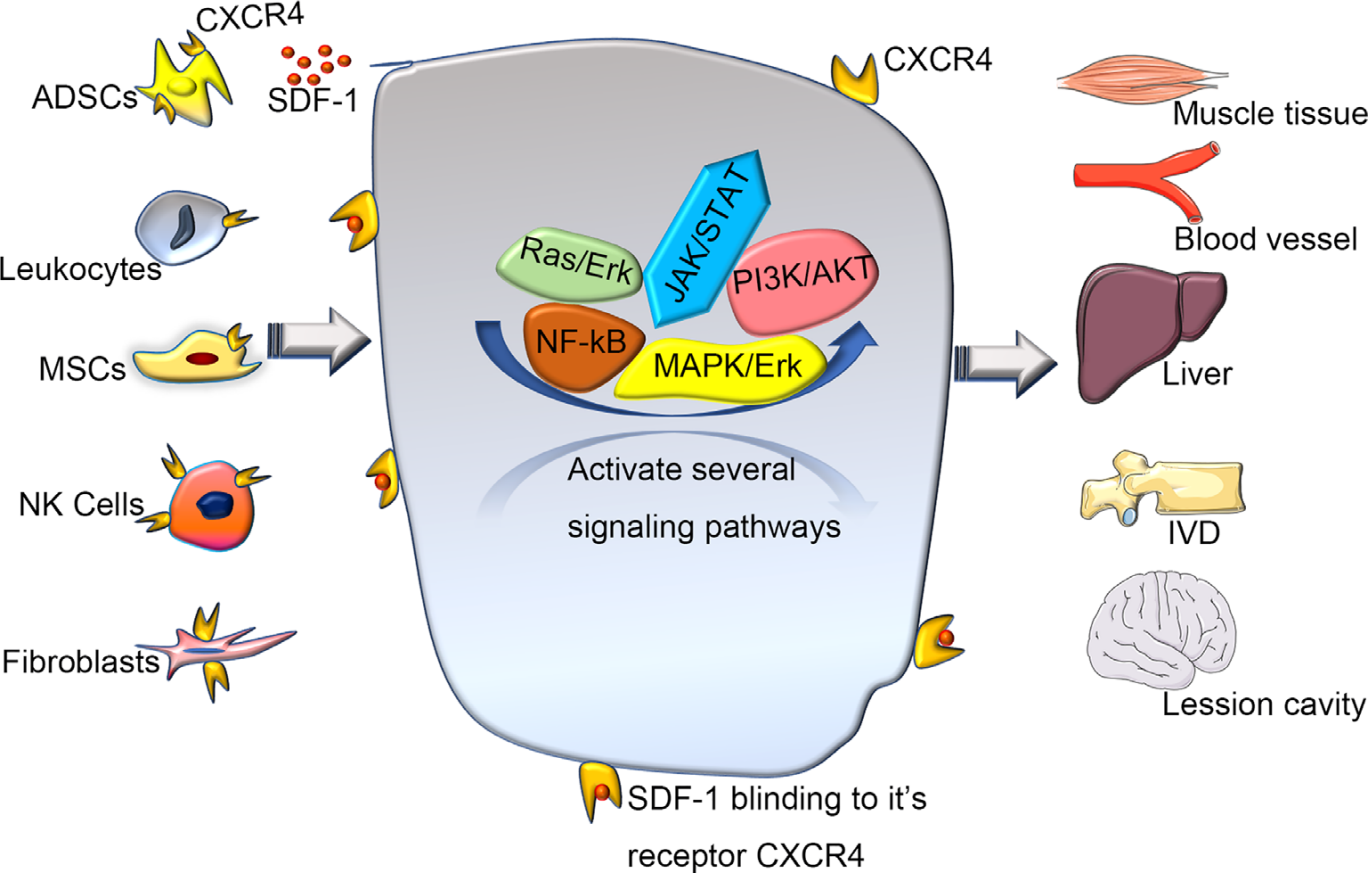
The various of wound healing *via* CXCR4 signaling pathway. Various cells expressing CXCR4 activate and participate in a series of signal networks to promote wound repair and regeneration. Recently, regeneration experiments based on the CXCR4 signaling pathway have used various stem cells as intermediate carriers. Transplanting stem cells that overexpress CXCR4, can promote repair and regeneration better through a directly or coordinate pattern. SDF-1, stromal cell derived factor-1; ADSCs, adipose tissue-derived stem cells; MSCs, mesenchymal stem cells; NK Cells, Natural Killer Cells; IVD, intervertebral disc.

CXCR4-induced migration is regulated by multiple signaling pathways. Many studies have reported that the MAPK and PI3K/Akt pathways are involved in cytokine- or chemokine-induced migration of various cell types (21, 26, 83, 84), PI3K may have the more important role in cytokine- or chemokine-induced migration (68). In some cell systems, PI3-kinase–dependent signaling contributes to several aspects of the migratory machinery, including signal amplification, gradient sensing, actin reorganization and thus, cell motility (41, 85). The activation of phospholipase C (PLC), diacylglycerol-dependent protein kinase C (PKC) and calcium mobilization by chemokines have also been proposed to regulate cell adhesion and migration (86, 87). Also, CXCR4-mediated migration can be enhanced by inducing PLC/PKC-Ca2+ signalling (88). Preclinical data show that bone-marrow MSCs can promote osteosarcoma growth *via* PI3K/Akt and Ras/Erk intracellular cascades and may enhance metastasis *via* CXCR4 signaling (89) (**Figure 3**).

## CXCR4 Regulates Cellular Apoptosis During Tissue Regeneration

Apoptosis regulated by CXCR4 plays a major biological modification role in tissue regeneration. The process of tissue regeneration includes physiological processes such as cell proliferation and chemotaxis, and of course, cell apoptosis. Like cell growth, development, and proliferation, apoptosis plays an important role in the life cycle of cells. The elimination of apoptotic cells caused by the inflammatory environment is a key step in wound healing (90, 91). CXCR4 is related to cell apoptosis. Downregulated CXCR4 can induce cell apoptosis by inhibiting the PI3K/Akt/NF-κβ signaling (92). Also, the activation of Akt can modulate proapoptotic or antiapoptotic proteins *via* transcriptional or posttranscriptional modes (93) (**Figure 1**). Binding of SDF-1 to CXCR4 and CXCR7 leads to anti-apoptotic signaling *via* Bcl-2 as well as promotion of the epithelial to interstitial transition through the Rho-ROCK pathway and alterations in cell adhesion molecules (72). Overexpression of miR-9-5p inhibited MAPK/ERK and PI3K/AKT/mTOR pathways by inhibiting CXCR4, thereby reducing high sugar induced human umbilical cord endothelial cell conversion (94). In both *in vitro* and *in vivo* experiments, Abraham et al. (95) showed that the CXCR4 antagonist BL-8040 could induce cells apoptosis. This apoptosis was mediated by upregulation of miR-15a/miR-16-1, thus resulting in downregulation of the target genes B-cell lymphoma 2, myeloid cell leukemia 1 and cyclin-D1. The authors showed that BL-8040 can induce apoptosis by inhibiting survival signals through the AKT/ERK pathway. Moreover, miR-146a can downregulate CXCR4 expression dose- and time-dependently. Phenotype experiments revealed that miR-146a mimics can inhibit cell proliferation and cell migration and promote apoptosis by targeting CXCR4 (96). Petri’s experiment found that the ratio of transforming growth factor beta/interleukin 6 (TGF-β/IL-6) ratio is related to the expression of CXCR4 (8). In addition, Arck and Hecher et al. (97) showed that the two cytokines TGF-β and IL-6 are involved in inducing CXCR4 as well as differentiating lymphocytes while driving senescence.

## Conclusion and Future Perspectives

Tissue regeneration and its application in regenerative medicine have always been urgently needed, and much energy and time have been invested in those topics. However, the form and function of tissue regeneration achieved is incomplete. CXCR4 clearly plays a pivotal role in tissue regeneration, and overexpression of CXCR4 in various stem cells can improve the survival of stem cell transplantation and induce various wound regeneration processes. At the same time, the activation of the CXCR4 signaling pathway can induce corresponding changes in multiple signaling pathways, then participate in the entire process of wound healing. This is the basis of the pleiotropic effects of CXCR4. Inhibiting the expression of CXCR4 can promote wound healing in a scarless manner during later phases of inflammation. To date, although a large body of studies deals with promoting wound healing through SDF-1/CXCR4 signaling pathway in small animals such as rats, limited progress has been made in neither large animals nor humans. Given that CXCR4 plays different roles during different periods of wound healing and regeneration, future therapies promise to achieve scarless wound healing by transplanting CXCR4 over-expressing stem cells into the wound post-injury and administrating CXCR4 inhibitors such as AMD3100 during the later phase of the wound healing. And, transition of research subjects from small animals to humans is expected to achieve the goal of perfect wound repair in the future. And, Research into wound healing may well focus on how to promote or inhibit CXCR4 signaling to differentiate stem cells into different tissues and how to provide better function in new tissue. Therefore, further study of CXCR4 may be an important way to more readily achieve tissue regeneration. Regulating the SDF-1/CXCR4 signal axis may provide a feasible method for realizing optimal wound repair and tissue regeneration.

## Author Contributions

HC and GL conceived and drafted the manuscript. YLiu, SJ, YLi, JX, XS, XF, and BL discussed the concepts of the manuscript. HC, YLiu, and SJ drew the figures. LZ and HG participated in the revision of the article. WZ drew the second picture and participated in the revision of the article. All authors contributed to the article and approved the submitted version.

## Funding

This work was supported in part by National Key Research and Development Plan (2018YFC1105704, 2017YFC1103304, 2016YFA0101000, 2016YFA0101002), the National Nature Science Foundation of China (81871569, 81830064, 81721092), the CAMS Innovation Fund for Medical Sciences (CIFMS, 2019-I2M-5-059) and the Military Medical Research and Development Projects (AWS17J005, 2019-126).

## Conflict of Interest

The authors declare that the research was conducted in the absence of any commercial or financial relationships that could be construed as a potential conflict of interest.
